# A Prospective Randomized Study Comparing Glenohumeral and Subacromial Corticosteroid Injections in the Management of Primary Frozen Shoulder

**DOI:** 10.7759/cureus.104057

**Published:** 2026-02-22

**Authors:** Namkumpeung Lungalang, Kumar Shantanu, Devarshi Rastogi, Brij Mohan Patel, Ashish Kumar, Shatakshi Pant

**Affiliations:** 1 Department of Orthopaedic Surgery, King George's Medical University, Lucknow, IND

**Keywords:** adhesive capsulitis, constant-murley score, corticosteroid therapy, glenohumeral injection, primary frozen shoulder, range of motion, subacromial injection

## Abstract

This prospective randomized comparative study evaluated the clinical efficacy of glenohumeral (GH) versus subacromial (SA) corticosteroid injections in managing primary frozen shoulder. The study was conducted at a tertiary care center in North India between 2023 and 2025. Sixty patients aged 20-70 years with stage 1 primary frozen shoulder, unresponsive to at least six weeks of conservative treatment, were included. Participants were randomly allocated to receive either a GH or SA injection containing triamcinolone, lidocaine, and saline. Each followed a standardized home-based exercise program. Outcomes were assessed at baseline and at 4, 8, and 12 weeks post-injection using the visual analogue scale (VAS), Constant-Murley score, and shoulder range of motion (ROM) parameters. These included forward (FWD) flexion, abduction, and internal and external rotation. Both GH and SA injections resulted in statistically significant improvements across all parameters over time (p < 0.0001). Comparative analysis revealed no significant difference between groups in pain relief, activities of daily living (ADLs), or internal rotation. However, GH injections showed significantly superior improvement in FWD flexion, abduction, and external rotation compared with SA injections. The findings suggest that both injection sites are effective for pain relief and functional improvement in primary frozen shoulder. Intra-articular GH injections may offer greater benefits for restoring specific shoulder movements, particularly those most affected by capsular fibrosis.

## Introduction

Frozen shoulder, also known as adhesive capsulitis, is a common musculoskeletal condition. It is characterized by progressive pain and a marked restriction in both active and passive range of motion (ROM) of the glenohumeral (GH) joint [1]. This disorder mainly affects individuals in middle age. It is more common in patients with diabetes, where stiffness tends to be more severe and requires proactive management [2,3]. The underlying pathology involves chronic inflammation, leading to fibrotic contracture of the GH joint capsule. The anterosuperior capsule, including the rotator interval and the coracohumeral ligament, is especially affected [4,5]. The fibrotic change leads to a predictable loss of motion. External rotation is most affected, followed by abduction, then internal rotation [6,7]. The exact cause of primary frozen shoulder is unknown. However, the condition is self-limiting, though it can result in prolonged disability and discomfort [1,8]. Conservative management includes physical therapy, oral anti-inflammatory drugs, and various injection therapies [2,3]. Of these, corticosteroid injections are widely used and effective due to strong anti-inflammatory effects [8,9]. Corticosteroids reduce inflammation in the joint capsule, which decreases pain and may improve mobility [6-8]. Despite their popularity, the optimal site for corticosteroid administration remains debated [9-11].

Historically, two primary injection sites have been investigated: the GH (intra-articular) space and the subacromial (SA) space [6,8]. The GH joint is the main articulation of the shoulder, and its capsule is directly implicated in the fibrotic changes characteristic of frozen shoulder. Therefore, intra-articular injection aims to deliver corticosteroids directly to the primary site of pathology [6-8]. In contrast, the SA space lies above the rotator cuff tendons. It is often associated with impingement syndrome, a condition that can present with symptoms similar to those of frozen shoulder but involves different anatomical structures [6,8]. Although the SA space is distinct from the GH joint, some theories suggest that inflammation in this region may contribute to or exacerbate the symptoms of frozen shoulder, thereby justifying SA injections [8]. Numerous prospective, randomized controlled trials and meta-analyses have been conducted to compare the efficacy of GH and SA corticosteroid injections in the short term [6-8,11]. These studies typically evaluate outcomes based on pain reduction measured by the visual analogue scale (VAS), improvements in various shoulder ROM parameters (e.g., external rotation, abduction), and functional scores such as the Constant score or the Simple Shoulder Test [6-8]. The results of these comparative analyses are crucial for guiding clinical practice and optimizing treatment protocols for patients with primary frozen shoulder. The goal of such research is to determine whether one injection site offers superior short-term pain relief, functional improvement, or a more rapid recovery trajectory, thereby informing evidence-based treatment decisions. Beyond single-site injections, more recent research has also begun to explore the efficacy of specific intra-articular locations, such as the rotator interval, and the potential benefits of multisite injection strategies, further complicating the decision-making process for optimal treatment [4,5,12,13].

The present study compared the clinical efficacy of steroid injections in patients with primary frozen shoulder, according to the injection site (GH joint vs. SA space), using a prospective, randomized comparison design. The aim was to evaluate post-injection pain and function using shoulder ROM and the Constant-Murley score. We hypothesized that clinical outcomes of GH steroid injections in patients with primary frozen shoulder would be better than those of SA injections.

## Materials and methods

Study design

This randomized comparative study was conducted at the Department of Orthopaedic Surgery, King George's Medical University, between 2023 and 2025. The Institutional Ethics Committee approved the study.

Study population

**Figure 1:**
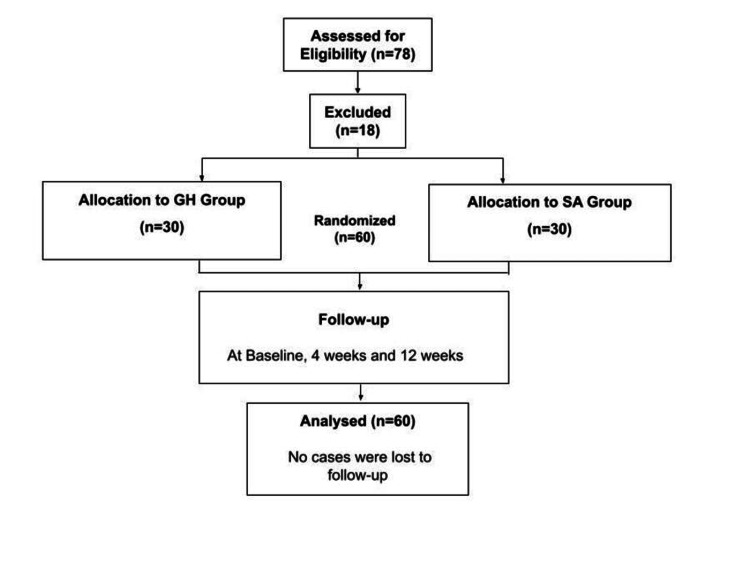
CONSORT flow diagram of patient enrollment, randomization, allocation, follow-up, and analysis. This flow diagram illustrates the progression of participants through the randomized controlled study. A total of 78 patients were assessed for eligibility, of whom 18 were excluded. Sixty patients were randomized equally to receive either glenohumeral (GH) or subacromial (SA) corticosteroid injections (30 in each group). All participants completed follow-up assessments at baseline, 4 weeks, and 12 weeks, and all randomized patients were included in the final analysis.

Subjects with primary frozen shoulder were selected according to inclusion and exclusion criteria. Based on an estimated prevalence of 3% and a 95% CI, the minimum calculated sample size was 45 participants. Accounting for an anticipated 25% dropout rate, the final sample size was increased to 60 patients, with 30 participants allocated to each group. The study included cases aged 20-70 years who were diagnosed with primary frozen shoulder and had normal radiographic findings of the affected shoulder. Participants in the study were excluded if they had secondary causes of frozen shoulder, including rheumatic diseases, infectious arthritis, tumors, rotator cuff disease, osteoarthritis of the GH joint, fibromyalgia, shoulder fractures, or other infections. Additionally, patients who had received corticosteroid injections in the affected shoulder within the previous three months were also excluded from the study. All samples had passive motion restriction of the GH joint greater than 30° in at least two motion planes (abduction and forward (FWD) flexion < 100°, external rotation < 20°, or internal rotation < 30°). Stage 1 was defined according to the Hannafin and Chiaia criteria, with pain duration <9 months and a VAS score for shoulder pain ≥3. Randomization of cases was conducted using an automated Excel (Microsoft Corp., Redmond, WA, USA) list. Patients were divided into two groups based on the injection site. All enrolled subjects had experienced no improvement in their frozen shoulder symptoms despite receiving conservative management, such as medication and physiotherapy, for a minimum of six weeks (Figure 1).

Surgical procedure

A posterior approach was used for injections into the GH joint, while a lateral approach was utilized for the SA space. After preparing the area aseptically, we located the soft spot approximately 2-3 cm inferior to the posterolateral corner (acromial angle) of the acromion. This spot corresponds to roughly two finger-breadths inferior and one finger-breadth medial to the posterior acromion. The needle was then inserted at this location and directed anteriorly toward the coracoid process to access the GH joint. For the SA space, we palpated the lateral edge of the acromion and inserted the needle 2-3 cm inferior to its lateral border, directing it superiorly and medially into the SA space between the acromion and the humeral head. In this study, all participants in both treatment groups received an injection containing 1 mL of triamcinolone (40 mg), 4 mL of 2% lidocaine, and 4 mL of normal saline for therapeutic purposes. To manage pain, patients were prescribed nonsteroidal anti-inflammatory drugs (NSAIDs) and analgesics. Furthermore, a self-exercise program was provided, focusing on gentle active-assistive or passive exercises designed to promote shoulder mobility. These exercises included FWD flexion, abduction, external rotation, adduction, and the sleeper stretch. Each stretch is recommended to be held for 5-10 seconds and can be gradually performed up to 10 repetitions per set, with 3-5 sets per day. Patients were advised to gently stretch their shoulders to the point of comfort, avoiding any strengthening exercises until their shoulder pain had subsided. To evaluate any adverse effects of steroid injection, blood sugar levels of diabetic patients were randomly checked before injection and rechecked four weeks after injection.

Outcome measurements

All data (before injection and at 4, 8, and 12 weeks after injection) were collected prospectively. Pain scores were assessed using the VAS and the Constant-Murley score for subjective function at each study visit. General perception or overall pain over 24 hours was determined using the VAS at each follow-up interview. 

Data management and analysis

For the statistical analysis, the collected data were entered into a Microsoft Excel sheet (2018-2019 version). Descriptive statistics, including percentages, means, standard deviations, and p-values, were calculated. When necessary, the data were presented in tables and graphs using GraphPad Prism 5 (Dotmatics, Boston, MA< USA). A one-way ANOVA was conducted to compare recovery at three different follow-up times for each activity parameter. Additionally, a two-way ANOVA was performed to assess the mean values of all three follow-ups for each individual activity parameter between the two study groups, utilizing the Row factor p-value.

## Results

The two groups were comparable with respect to age, sex distribution, baseline pain scores, and ROM parameters (Table 1). At various follow-up time points, both injection sites exhibited significant differential effects on all six activity parameters: activities of daily living (ADLs) (Table 2), mean pain point (Table 3), FWD flexion (Table 4), abduction (Table 5), external rotation (Table 6), and internal rotation (Table 7), with excellent p-values of 0.0001 for each parameter.

**Table 1:** Baseline characteristics of the study participants. GH: glenohumeral, SA: subacromial.

Variable	GH group (n = 30)	SA group (n = 30)
Age (years)	49.2 ± 5.24	46.8 ± 6.45
Male	8 (26.7%)	5 (16.7%)
Female	22 (73.3%)	25 (83.3%)
Dominant side affected	26 (86.7%)	25 (83.3%)
Duration of symptoms (days)	55 ± 6.25	63 ± 3.18
Diabetic patients	16 (53.3%)	11 (36.7%)

**Table 2:** Comparison of activities of daily living (ADL) scores following glenohumeral and subacromial corticosteroid injections in patients with primary frozen shoulder. Mean ADL scores were assessed at baseline, 4 weeks, and 12 weeks after injection. Two-way ANOVA showed a significant improvement in ADL scores over time in both groups (column factor p = 0.0022**), with no statistically significant difference between the two injection sites (row factor p = 0.2020). Values represent mean scores. **p < 0.01.

Site of injection	Mean activities of daily living		
	Baseline	4 weeks	12 weeks
Glenohumeral	10.04 ± 2.35	15.60 ± 2.84	18.32 ± 2.27
Subacromial	9.88 ± 2.51	14.72 ± 2.32	18.08 ± 2.45

**Table 3:** Comparison of pain scores following glenohumeral and subacromial corticosteroid injections in patients with primary frozen shoulder. Mean pain scores were recorded at baseline, 4 weeks, and 12 weeks post-injection. Two-way ANOVA demonstrated a significant improvement in pain scores over time in both groups (column factor p = 0.0006***), while no statistically significant difference was observed between the injection sites (row factor p = 0.3061). Values represent mean scores. ***Highly significant (p < 0.001).

Site of injection	Mean pain point		
	Baseline	4 weeks	12 weeks
Glenohumeral	4.00 ± 1.32	8.92 ± 1.41	13.00 ± 1.08
Subacromial	4.04 ± 1.46	8.76 ± 1.79	12.68 ± 1.28

**Table 4:** Comparison of forward flexion following glenohumeral and subacromial corticosteroid injections in patients with primary frozen shoulder. Mean forward flexion scores were recorded at baseline, 4 weeks, and 12 weeks post-injection. Two-way ANOVA demonstrated a significant improvement over time in both groups (column factor p = 0.0094**), with a statistically significant difference favoring the glenohumeral injection group (row factor p = 0.0429*). Values represent mean scores. *Significant (p < 0.05). **p < 0.01.

Site of injection	Mean forward flexion		
	Baseline	4 weeks	12 weeks
Glenohumeral	6.08 ± 1.35	7.6 ± 1.73	8.72 ± 1.55
Subacromial	5.28 ± 0.98	7.2 ± 1.29	7.84 ± 1.52

**Table 5:** Comparison of shoulder abduction following glenohumeral and subacromial corticosteroid injections in patients with primary frozen shoulder. Mean abduction scores were assessed at baseline, 4 weeks, and 12 weeks after injection. Two-way ANOVA showed a significant improvement over time in both groups (column factor p = 0.0013***), with a statistically significant difference between injection sites favoring the glenohumeral group (row factor p = 0.0036**). Values represent mean scores. **p < 0.01. ***Highly significant (p < 0.001).

Site of injection	Mean abduction		
	Baseline	4 weeks	12 weeks
Glenohumeral	5.83 ± 1.55	7.58 ± 1.77	8.17 ± 1.55
Subacromial	4.92 ± 1.18	6.75 ± 1.54	7.42 ± 1.50

**Table 6:** Comparison of external rotation following glenohumeral and subacromial corticosteroid injections in patients with primary frozen shoulder. Mean external rotation scores were measured at baseline, 4 weeks, and 12 weeks post-injection. Two-way ANOVA demonstrated a significant improvement over time in both groups (column factor p = 0.0006***), with a statistically significant difference between injection sites favoring the glenohumeral group (row factor p = 0.0027**). Values represent mean scores. **p < 0.01. ***Highly significant (p < 0.001).

Site of injection	Mean external rotation		
	Baseline	4 weeks	12 weeks
Glenohumeral	4.16 ± 0.55	6.25 ± 0.90	7.92 ± 1.22
Subacromial	3.25 ± 1.29	5.25 ± 1.54	6.83 ± 1.55

**Table 7:** Comparison of internal rotation following glenohumeral and subacromial corticosteroid injections in patients with primary frozen shoulder. Mean internal rotation scores were assessed at baseline, 4 weeks, and 12 weeks after injection. Two-way ANOVA showed a significant improvement over time in both groups (column factor p = 0.0028**), while no statistically significant difference was observed between the two injection sites (row factor p = 0.2943). Values represent mean scores. **p < 0.01.

Site of injection	Mean internal rotation		
	Baseline	4 weeks	12 weeks
Glenohumeral	2.76 ± 1.20	5.20 ± 1.29	6.16 ± 1.28
Subacromial	2.80 ± 1.29	4.88 ± 1.42	6.00 ± 1.15

When evaluating the comparative effects of two injection sites on various parameters during follow-up intervals, we found that three activity parameters, such as pain relief, ADLs, and internal rotation, showed no significant difference between the injection sites. Both sites had a p-value of 0.3061 (row factor p-value from the two-way ANOVA) regarding pain relief. For ADLs and internal rotation, the p-values were 0.2020 and 0.2943, respectively.

In contrast, three other parameters, namely, FWD flexion, abduction, and external rotation, demonstrated significant differences between the two injection sites. The p-value for FWD flexion was 0.0429, while for abduction and external rotation, the values were 0.0036 and 0.0027, respectively.

## Discussion

Our study conducted a thorough comparative analysis of GH and SA corticosteroid injections for the treatment of primary frozen shoulder. We discovered both similarities and differences in their effectiveness across various activity parameters. Specifically, both injection sites resulted in significant improvements in all six assessed areas: pain relief, ADLs, FWD flexion, abduction, external rotation, and internal rotation, with a p-value of less than 0.0001 for each parameter. However, a comparative analysis of the two injection sites at different time intervals revealed important distinctions. Regarding pain relief, ADLs, and internal rotation, the study found no significant differences between the injection sites. The p-values were 0.3061 for pain relief, 0.2020 for ADL, and 0.2943 for internal rotation, all based on the row factor p-value from a two-way ANOVA [10]. This indicates that both intra-articular and SA injection approaches provide similar therapeutic benefits for these outcomes. This finding is particularly intriguing when compared to existing literature. A meta-analysis by Shang et al. indicated that intra-articular corticosteroid injections generally provided better clinical outcomes than SA injections for pain relief and improvement in ROM in adhesive capsulitis [11]. Similarly, Chen et al. found superior efficacy of intra-articular injections, as evidenced by greater VAS score reduction, improved ROM (including external rotation and abduction), and better Constant scores, compared with SA injections [7]. The apparent discrepancy, especially concerning pain relief, might stem from differences in study design, patient populations, injection techniques (e.g., ultrasound guidance), or specific corticosteroid formulations and dosages. For instance, the study by Oh et al. (2011) observed that while GH injections showed statistically significant better improvements in VAS score at three weeks, by six weeks, no significant differences were observed between GH and SA groups, suggesting that the initial advantage of GH injections in pain relief might be short-lived or converge over time [8]. However, the present results clearly demonstrated significant differences in the effects of the two injection sites on FWD flexion, abduction, and external rotation [10]. The p-value differences were 0.0429 for FWD flexion, 0.0036 for abduction, and 0.0027 for external rotation. These significant differences imply that one injection site is superior to the other for improving these specific ROMs. Given that primary frozen shoulder is fundamentally characterized by a fibrotic contracture of the GH joint capsule, leading to restricted ROM, especially external rotation and abduction [2,3,14], direct intra-articular injections are hypothesized to improve joint mobility by delivering corticosteroids to the site of capsular inflammation and fibrosis [15].

Several studies support the notion that intra-articular injections are more effective for improving ROM. The meta-analyses by Chen et al. and Shang et al. both reported superior outcomes for intra-articular injections in improving ROM, including abduction and external rotation [7,11]. Furthermore, a randomized controlled trial by Sun et al. suggested that a single injection into the rotator interval, an intra-articular location, could yield better effects in terms of pain, ROM, and function than injections into the SA space for early-stage frozen shoulder [4]. The rotator interval is a critical anatomical area often implicated in the pathogenesis of adhesive capsulitis [15]. This aligns with the observation that parameters directly related to GH joint mechanics, such as FWD flexion, abduction, and external rotation, show differential responses between the two injection sites in the current study. The finding that certain parameters, such as pain relief and ADL, do not show significant differences between injection sites, whereas specific ROMs do, highlights the complexity of assessing treatment efficacy in frozen shoulder. It suggests that while both approaches may alleviate generalized discomfort and improve functional daily activities to a similar extent, their impact on restoring specific joint movements might differ. This could be due to the varied pathophysiology of frozen shoulder, which involves both inflammation and fibrosis within the joint capsule, and potentially inflammatory processes in surrounding periarticular structures. Corticosteroids effectively address inflammation, but mechanical restriction due to fibrotic changes may require more direct intervention, such as precise intra-articular delivery to the fibrosed capsule. Recent research has also explored more targeted intra-articular approaches and multisite injections. For example, a meta-analysis by Arrambide-Garza et al. compared ultrasound-guided corticosteroid injections via the rotator interval versus the posterior approach and found potentially greater efficacy of rotator interval injections in improving pain and ROM at six weeks [16]. Additionally, studies by Saud et al. (2023) and Koraman et al. (2021) demonstrated that multisite injections around the shoulder could be more effective than a single GH injection for pain relief, ROM, and functional scores, suggesting that a more comprehensive approach might yield superior outcomes [12,17]. These advancements indicate a growing understanding that the optimal treatment strategy might involve not only the general site but also the specific intra-articular location, or even a combination of sites, to address the multifactorial nature of frozen shoulder.

In conclusion, the presented results indicate comparable effects on pain relief, ADL, and internal rotation, but significant differences in FWD flexion, abduction, and external rotation, aligning partially with the broader literature. While some meta-analyses suggest overall superiority of intra-articular injections, particularly for improvements in ROM, the current study provides granular insights into specific parameters where differences are pronounced. This differentiation underscores the importance of precise injection targeting for specific functional outcomes, potentially advocating for intra-articular approaches when restoring FWD flexion, abduction, and external rotation is a primary goal, while acknowledging that both sites may provide similar benefits for pain and general daily activities in the short term. Further research is needed to elucidate the long-term implications and to optimize injection protocols based on the specific clinical presentation and patient-reported outcomes.

The present study is limited by a relatively small sample size and a short follow-up duration of 12 weeks, which precludes assessment of long-term outcomes and recurrence. Injections were performed using landmark-guided techniques rather than ultrasound guidance, which may have affected the accuracy of corticosteroid placement. Adherence to the prescribed home-based exercise program was not objectively monitored, potentially influencing outcomes. Additionally, inclusion was restricted to patients with early-stage primary frozen shoulder, limiting generalizability to later disease stages.

## Conclusions

Both groups showed significant relief across all six movement parameters when evaluated separately at the GH and SA injection sites throughout the follow-up. But when both injection site groups were compared with each other, relief in only three out of six movement parameters, namely, FWD flexion, abduction, and external rotation, was significantly higher in the GH group than in the SA group.
